# A Pickering Emulsion Route to Swimming Active Janus Colloids

**DOI:** 10.1002/advs.201700528

**Published:** 2017-12-01

**Authors:** Richard J. Archer, Andrew J. Parnell, Andrew I. Campbell, Jonathan R. Howse, Stephen J. Ebbens

**Affiliations:** ^1^ Department of Chemical and Biological Engineering The University of Sheffield Sheffield S1 3JD UK; ^2^ Department of Physics and Astronomy The University of Sheffield Sheffield S3 7RH UK; ^3^ Department of Chemistry University of Sheffield Sheffield S3 7HF UK

**Keywords:** active colloids, catalysis, Pickering emulsions

## Abstract

The field of active colloids is attracting significant interest to both enable applications and allow investigations of new collective colloidal phenomena. One convenient active colloidal system that has been much studied is spherical Janus particles, where a hemispherical coating of platinum decomposes hydrogen peroxide to produce rapid motion. However, at present producing these active colloids relies on a physical vapor deposition (PVD) process, which is difficult to scale and requires access to expensive equipment. In this work, it is demonstrated that Pickering emulsion masking combined with solution phase metallization can produce self‐motile catalytic Janus particles. Comparison of the motion and catalytic activity with PVD colloids reveals a higher catalytic activity for a given thickness of platinum due to the particulate nature of the deposited coating. This Pickering emulsion based method will assist in producing active colloids for future applications and aid experimental research into a wide range of active colloid phenomena.

## 1 Introduction

Micrometer and nanoscale synthetic swimming devices show enhanced displacements far exceeding Brownian motion by exploiting localized catalytic reactions. Such devices have generated interest due to potential applications ranging from microfluidic transport in lab‐on‐a‐chip devices,^1^, ^2^, ^3^ rapid environmental decontamination,^4^ and directed drug delivery.^5^ As evidence of the growing scope for real‐world applications for these devices, a recent report showed improved delivery of a model drug in vivo.^6^ In addition, self‐motile swimming devices also provide a route to experimentally verify a wide range of phenomena that have been predicted for colloidal systems. One particular area of interest is the potential for high volume fractions of interacting motile colloids to display a rich variety of emergent behavior, including self‐organizing effects such as clustering.^7^ However, despite this attention, the current methods by which synthetic catalytic swimming devices are manufactured remain cumbersome and have significant drawbacks, which limit the viability of proposed applications, and prevent extensive experimental research effort into active colloid phenomena. Prominent, widely studied examples of synthetic swimming devices include bimetallic rods, consisting of connected catalytically active and inactive segments,^8^ microrockets, consisting of rolled‐up microtubes with catalyst coating the interior walls,^9^ and Janus particles, consisting of spherical colloids where one hemisphere is catalytically active.^10^ Platinum is used as the catalytically active material in the majority of the reported examples due to its ability to perform the rapid room‐temperature decomposition of hydrogen peroxide, and consequently produce motion via either phoretic^11^ or bubble release mechanisms.^12^ A key feature of the three device types is that motion production requires a specific distribution of catalyst, particularly for nanorod and Janus devices that move via self‐phoresis where the mechanism is critically reliant on gradients which can only be generated by having an asymmetric catalyst distribution. Taken together, the requirement for both metallization and asymmetry generation has resulted in the current manufacturing methods being typically cumbersome and low yielding, owing to a reliance on lab‐based physical vapor deposition (PVD) techniques to deposit the catalyst. For the aforementioned examples, bimetallic rod synthesis requires a combination of PVD and electroplating into porous membranes for each required metal, microrockets require PVD of multiple metals onto sacrificial polymer films which when etched away cause the metal films to roll into the required tubular structure, while active Janus colloids require platinum PVD onto spherical colloids.^10^, ^13^, ^14^ The PVD requirement restricts all these processes to 2D planar batch fabrication, and the requirement during PVD for high vacuum environments creates scalability issues and high energy demands to vaporize the source material. PVD is currently hard to implement as a continuous process as it requires transfer of material for coating from ambient to vacuum conditions. Additionally, unless scrupulously clean, vacuum chambers are subject to introducing surface hydrocarbon contamination which can impair reactivity and require cleaning stages. Moreover, PVD requires both localization of colloids onto a solid support with a degree of control (e.g., spin‐coating) to avoid shadowing, and finally transfer of colloids back into the solution phase, which is hard to accomplish efficiently and can introduce contamination. Another general drawback for PVD techniques is the difficulty in maintaining stoichiometry when evaporating compound materials such as metal oxides, often requiring the introduction of reactive gases into the chamber. These metal oxide materials have been suggested as alternative more versatile catalysts for powering self‐motile systems.^15^ The potential to develop an analogue of the solution‐based method we present here to also deposit metal oxides as Janus coatings overcomes the stoichiometry issues with PVD.

**Figure 1:**
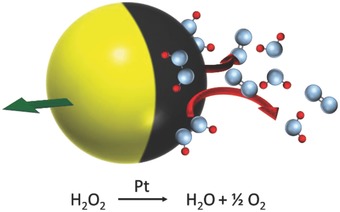
Schematic of a Janus swimming device showing the decomposition of hydrogen peroxide on the platinum‐covered hemisphere (black) to generate motion (green arrow).

The focus of this paper is to develop an alternative manufacturing route that can produce high yields of swimming devices, to both provide a viable route to scale‐up to meet the requirements of emerging applications, and to allow straightforward synthesis of lab‐scale batches of active colloids to aid investigation of active colloid phenomena. We focus specifically on spherical Janus colloids of the type shown in **Figure** **1**. This type of swimming device has been found to display a wide range of interesting colloidal phenomena, including autonomous guidance effects (gravitaxis,^16^ chemotaxis,^17^ and boundary steering),^18^ and has also been the subject of many currently untested theoretical proposals for high volume fraction collective phenomena.^19^ An advantage of Janus spheres as a system to explore emergent behavior is that they move without issuing bubbles and so their interactions via chemical “wakes” and hydrodynamics are amenable to being analyzed and experimentally observed, whereas nanotubes produce considerable convective flow due to bubble release.^20^ In addition, in contrast to nanorods, Janus spheres can also be studied in 3D bulk solution as their body can be neutrally buoyant, whereas metallic nanorods undergo rapid sedimentation.

For current Janus sphere synthesis, asymmetry is introduced due to the directionality of the platinum deposition process relative to a planar array of colloids, resulting in the underside of the colloid not receiving any platinum. Against this background, the aim of this study is to instead chemically deposit platinum on colloids asymmetrically in solution to give a low‐energy, scalable alternative route to micrometer‐scale self‐motile active colloids. The strategy we use is to partially mask colloids before performing solution phase deposition of platinum. The masking is achieved by a Pickering emulsion technique, where the colloidal bodies are trapped between an oil and water interface which on cooling solidifies the oil phase to create an impermeable barrier encompassing the oil submerged portion of the body and allowing the protruding portion to be selectively modified.^21^, ^22^

This study uses silica as the body material for the active colloids due to the ease in producing large‐scale monodisperse colloids with controllable diameters.^23^, ^24^ Silica offers other potential advantages in controllable porosity. Mesoporous silica is well documented and easily produced with tunable pore sizes through small modifications to the Stöber‐like processes used to make silica hard spheres.^25^ Silica has been extensively studied for its potential use in drug storage and delivery which is also a potential application for active Janus particles.^26^ Also the ready ability to functionalize silica surfaces via silanes^27^ assists the chemical deposition of platinum, but also will allow additional selective chemical modifications of the inactive side of the colloid.

While platinum films have been previously grown for other applications using electroless chemical deposition, such systems often rely on harsh chemicals such as hydrazine or elevated temperatures unsuitable for use with silica and wax masking.^28^ Instead, here we employ a seed and growth technique using a platinum salt precursor inspired by the previously documented ability to similarly grow gold shells on colloids.^29^ This method relies on the electrostatic adhesion of negatively charged metallic nanoparticles formed from rapid reduction of platinic acid using sodium borohydride to a positively charged amino propyl silane (APS) modified surface chemistry of the colloid.^30^ From these initial surface bound particles we attempt to control further addition to the surface through the slow reduction of platinic acid to its base metal using the mild reducing agent formaldehyde. This step aims to allow Pt^0^ to join the existing seeds, eventually forming thicker more continuous shells.^31^ The potential to control platinum thickness is of interest as this provides a possible route to control reactivity and morphology.^32^ A further advantage of this approach is that after platinum deposition and release from the Pickering emulsion, the catalytically inactive side of the silica colloid displays amine surface functionality, widely used for biological binding of proteins and antibodies which could potentially allow biological recognition and specific targeting applications.^33^, ^34^

To test the effectiveness of the chemically prepared active colloids, we benchmark catalytic activity, propulsion velocity, and rotational rate against equivalent conventionally PVD manufactured Janus particles.^35^ In addition, we compare batches of chemically prepared colloids with varying concentrations of platinum salt added during the seed growth stage of the protocol. In order to understand the effect of the growth conditions on active colloids performance, we also characterize the physical structure of the platinum coating using scanning electron microscopy (SEM) and atomic force microscopy (AFM).

## 2 Results and Discussion

### 2.1 Chemical Synthesis of Active Janus Colloids

**Figure 2:**
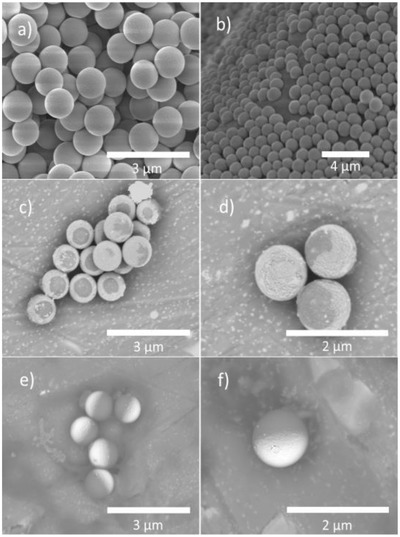
a) Monodisperse Stöber‐like synthesized silica of 1.1 µm average diameter. b) Surface section of solidified Pickering emulsion. c,d) Backscattered electron images of Pt‐coated silica after release from the wax. e,f) Backscattered electron images showing Pt‐coated silica prepared by PVD.

The synthesis of active Janus particles was carried out as described in the Experimental Section. Silica colloids grown by a Stöber‐like process (1.1 µm diameter, confirmed by Nanoparticle Tracking Analysis (NTA)) were surface modified with APS using the well‐known anhydrous reflux technique to introduce amine functionality across the entire particle surface. SEM was used to verify the surface texture and size of the silica colloids after this functionalization, **Figure** **2**a, and no change in the surface morphology or diameter could be detected, consistent with the expected molecular coating. Before solution deposition of platinum was attempted, these APS‐modified silica colloids were added to a molten wax in water Pickering emulsion. This stage is intended to partially mask the APS colloid surface. The emulsion formed with ≈84% yield with respect to mass of initial wax, the remaining 16% did not emulsify and instead coated the reaction vessel walls, this minor wax portion appeared to contain no embedded silica by SEM observations, nor was any significant quantity of silica colloids found outside of the emulsified wax after completion of the solidified emulsion. All non‐embedded silica colloids are removed during washing stages after the formation of the solidified wax, removing the potential for secondary non‐Janus functionalized particles. The resulting solidified and washed Pickering emulsion was then observed by SEM. The wax contained a densely packed arrangement of silica colloids partially embedded into the wax surface, Figure 2b. The embedded depth could only be observed in regions of lower colloidal coverage which revealed roughly one‐third of the colloid to be submerged into the wax (Figure 2b). While some variation in precise penetrative depth was noted, the approximate submerged fraction was highly consistent.

Having established that the masking stage required to introduce asymmetry had been successful, a variety of batches of colloids were chemically modified with platinum, using a two‐stage seeding and growth approach discussed in the Experimental Section. The first rapid reduction stage to generate the platinum seeds was carried out with a constant amount of platinum salt added (8.46 n mol cm^−2^), while the second growth stage was performed with a varying amount of platinic salt from 0 to 17 n mol cm^−2^. After completion of platinum growth, the colloids were separated by selectively dissolving the masking wax. The freed colloids were examined by SEM.

**Figure 3:**
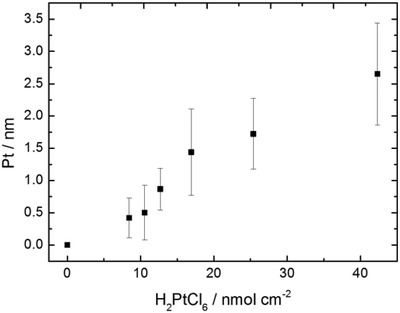
Platinum shell thickness of active colloids prepared through solution synthesis, calculated from sedimentation rate of the respective addition of total added platinic acid of 0, 8.46, 10.57,12.66, 16.92, 25.38, and 42.3 nmol cm^−2^ of total colloidal surface area.

Figure 2c,d shows backscattered SEM images of a typical batch of randomly orientated colloids (10.69 nmol of platinum per cm^2^ during growth stage). Due to the relatively heavy Pt nuclei backscattering electrons give higher signal intensity compared to the lowed mass silica body, so the surface distribution of platinum is seen as bright contrast. The heterogeneous contrast in the SEM images consequently indicates that platinum has been successfully deposited. The platinum is revealed to cover approximately two‐thirds of the spherical colloids in a continuous shell. This is consistent with the SEM observation of the wax embedded colloids. There is also some apparent variance in coverage and cap shape. Note that some colloids appear to have uniform platinum coverage, probably because the masked region is on the underside and so blocked from view. Each batch of colloids prepared with varying amounts of platinum salt added during the growth stage showed a qualitatively similar distribution of platinum after release from the Pickering emulsion. **Figure** **3** gives the experimentally determined average platinum thickness, determined through sedimentation velocity to find the increase in the average colloidal density, with any increase being assumed to come from platinum metal. A monotonic increase between platinum salt added and platinum metal deposited is found. Backscattered contrast in SEM images correlated with the expected deposited thickness, with brighter contrast for samples prepared with higher platinum salt concentrations.

For comparison, Figure 2e,f shows backscattered SEM images for randomly oriented silica colloids coated with a platinum hemisphere using the conventional PVD method. As expected, due to the line of sight shadowing effect that introduces the asymmetry in this preparation method, the cap is shown to be highly consistent with 50% platinum coverage, and the cap shape shows little variation. In addition, the platinum shells prepared by PVD appear qualitatively to be smoother than those prepared by chemical reduction of platinum precursors as evident by comparing Figure 2d and Figure 2f.

### 2.2 Catalytic Swimming Behavior

**Figure 4:**
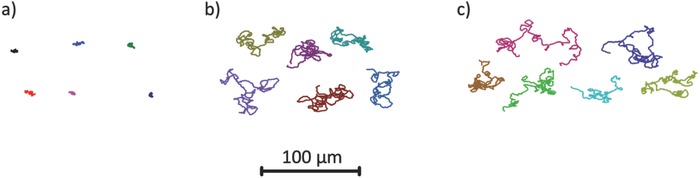
Representative *x*,*y* plots of particles displaying motion in 10% w/v H_2_O_2_, extracted from 30 s videos at 33 fps for a) silica colloids without catalyst and active colloids prepared from b) PVD to 10 nm thickness. c) Solution‐based growth of Pt from H_2_PtCl_6_ to a thickness of 2.65 nm.

Both PVD prepared colloids, and the chemical grown catalytically active Janus colloids were dispersed into a 10% wt/vol hydrogen peroxide solution and their motion was observed and recorded through optical microscopy. **Figure** **4**a shows example trajectories of colloids before coating with the catalyst in the hydrogen peroxide fuel; Figure 4b,c shows examples of trajectories for Janus colloids prepared by PVD (10 nm Pt coating thickness) and solution‐based synthesis (2.65 nm Pt shell thickness) in the hydrogen peroxide fuel. The nature of the trajectories is consistent with previous reports which show catalytically active colloids displaying short‐term ballistic‐type trajectories which are randomized due to rotational diffusion of the colloid as a result of the directionality of the propulsion vector being fixed in relation to the catalyst distribution.^36^ While the platinum distribution is different between the two synthesis methods, the resulting enhanced motion is strikingly similar: this indicates the clear potential to use the solution‐based method to make active colloids that will be suitable replacements for PVD prepared samples. This correspondence was not necessarily expected, as heterogeneity in the cap shape or patchiness of the solution grown platinum cap may have resulted in rapid spinning, or inconsistent behavior between different colloids.

Velocity (*V*) and rotational diffusion (τ_R_) coefficients were calculated for each batch of active colloids from the mean squared displacement (MSD) using Equation (1) (fitted to the quadratic section from 0 to 0.25 s, well below the rotational diffusion time) and Equation (2) (fitted to the linear section from 2 to 3 s, well above rotational diffusion time), respectively^10^

(1) $$\Delta L^{2}=4D\Delta t+V^{2}\Delta t^{2}$$

(2) $$\Delta L^{2}=\left(4D+V^{2}\tau_{R}\right)\Delta t-\frac{V^{2}\tau_{R}{}^{2}}{2}$$

**Figure 5:**
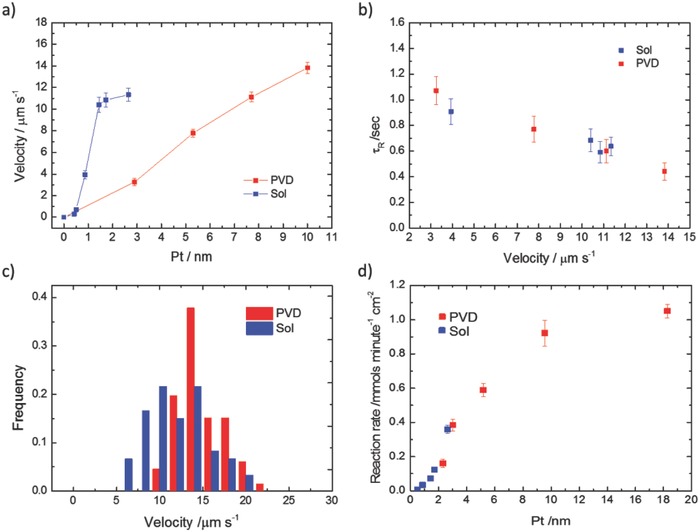
a) Translational velocity of active colloids prepared by PVD to platinum thicknesses of 2.9, 5.3, 7.7, and 10 nm and translational velocities of active colloids prepared by solution growth of platinum to thicknesses of 0.42, 0.50, 0.87, 1.44, 1.72, and 2.65 nm. b) Rotational diffusion of active colloids prepared by PVD to platinum thicknesses of 2.9, 5.3, 7.7, and 10 nm (red) and rotational diffusion of active colloids prepared by solution growth of platinum to thicknesses of 0.42, 0.50, 0.87, 1.44, 1.72, and 2.65 nm (blue). c) Histogram of relative velocity frequency at platinum thickness of 10 and 3.46 nm for PVD (red) and solution grown (blue) platinum. d) Reaction rate of decomposition of H_2_O_2_ per colloid active surface area for active Janus colloids prepared in solution (Sol) and by vapor deposition (PVD).

The same analysis was also applied to batches of colloids prepared using the PVD method with increasing platinum thickness. **Figure** **5**a shows the translational velocity comparison for PVD and solution prepared active colloids. It is apparent that the solution prepared colloids show a diminishing increase in translational velocity from 10.40 ± 0.68 µm s^−1^ at a thickness of just 1.44 nm. At over twice this thickness, the PVD prepared samples show a much slower velocity of ≈3 µm s^−1^, however these velocities increase monotonically up to 13.83 µm s^−1^ (±0.52 µm s^−1^) at 10 nm of platinum.

In addition, to this comparison of propulsion speed, it is also instructive to compare the rotational diffusion behavior that leads to randomization of the active colloids trajectories. Figure 5b shows the rotational diffusion time for the PVD and solution‐based preparations. In both cases, τ_R_ is close to the theoretical value (1.01 s at 21 °C), at low velocities but decreases with higher velocities. However, no evidence for regular spiraling behavior was seen in the trajectories or MSD plots, suggesting that the colloids were not producing a constant propulsive angular velocity vector, as is the case for PVD prepared Janus colloids with deliberately rotationally asymmetric catalytic activity.^37^ When a similar phenomenon was initially observed for PVD active colloids it was suggested to be caused by surface imperfections in the active cap. However, here we again find a striking similarity in the τ_R_ reduction as a function of velocity, despite the surface morphological differences between the two preparation techniques as seen between Figure 2d and Figure 2f. This suggests the phenomena may not be structural in origin. τ_R_ can be estimated using Equation (3), in which we see the rotational diffusion is affected by the temperature *T*, viscosity η, and the colloid radius *R*

(3) $$\tau_{R}=\frac{\left(8\pi \eta R^{3}\right)}{K_{B}T}$$

With the radius being a fixed and assuming the solution is a Newtonian fluid, therefore discounting the possibility of viscosity changes due to Janus colloid velocity generated shear, we can state that τ_R_ is possibly reduced due to localized changes in *T* and subsequently η through the exothermic decomposition of H_2_O_2_. However, as this would require a local increase of 20 °C to solely account for the observed decrease of τ_R_, we suspect additional contributing factors such as a structure unrelated reaction nonuniformity on the catalyst surface causing imbalances on the resulting flow field.

Figure 5c also gives translational velocity histogram data for the fastest PVD and solution‐based active colloids produced here. The solution‐based velocities show a slightly wider spread, which is not unexpected due to the variation in platinum distribution revealed by SEM.

In most models for phoretic self‐propulsion, there is a link between translational propulsion velocity and reaction rate,^32^ and so it is likely that features of the platinum coating that alter reaction rate will also effect propulsion speed. For the solution Pt‐coated colloids prepared here, varying the platinum salt concentration during the solution platinum growth stage can potentially control surface reactivity, and propulsion velocity. As shown in Figure 3, platinum salt concentration during the growth stage is measured to be proportionally related to the resulting colloidal platinum content, and the conditions selected were expected to produce a series of coatings in the range 0.40–2.65 nm. Figure 5d shows the rate of hydrogen peroxide decomposition as a function of this thickness estimate. It is clearly seen that decomposition rate increases monotonically with platinum thickness. Previous data for PVD catalytic coatings showing a similar monotonic increase in reaction rate up to 10 nm thickness are also plotted for comparison on Figure 5d.^32^ It is noted that both PVD and solution‐based colloids show a similar increase in reaction rate with catalyst thickness, and very similar reaction rates per area are seen in the region between 2 and 3.5 nm. For example, the solution prepared active colloids decompose hydrogen peroxide at a rate of 0.36 (±0.024) mmol min^−1^ cm^−2^ for 2.65 nm thickness where previous reports show PVD colloids decompose 0.38 (±0.033) mmol min^−1^ cm^−2^ for 3.02 nm thickness. This reaction rate comparison suggests that the solution‐based colloids are producing higher velocities despite having similar reactivity.

**Figure 6:**
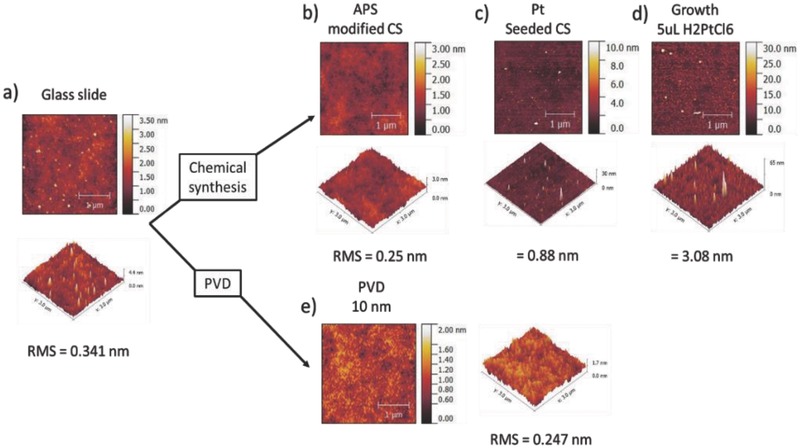
AFM measurement of a) unmodified glass surface, RMS 0.34 nm, b) glass surface modified with monolayer of APS, RMS 0.25 nm, c) APS modified glass washed with platinum seed solution, RMS 0.88 nm, d) Pt seeded surface after 3 days exposure to growth solution, RMS 3.08 nm, and e) glass surface after PVD of platinum (10 nm), RMS 0.25 nm.

To investigate further and quantify the roughness differences suggested in the SEM images of PVD and solution‐coated colloids, AFM was used to examine the surface morphology of a model flat silica surface sequentially subject to equivalent platinum coatings to those performed on the active colloids, **Figure** **6**. Quantification of root‐mean‐squared (RMS) surface roughness was also performed. First, considering the PVD deposition process, Figure 6a,b shows that silane modification of the surface with APS does not significantly change the surface topography or roughness, as is expected for monolayer assemblies. Figure 6c shows that the initial seeding stage of platinum deposition does change the surface topography, due to the surface adhesion of platinum nanoparticles. As the platinum is grown onto the seeds, the roughness increases further as shown in Figure 6d. (Final RMS roughness = 3.08 nm.) In comparison, PVD deposition of a 10 nm thick platinum film results in a RMS roughness of 0.25 nm, similar to that of the APS‐modified glass slide before seeding. Despite the much rougher particulate coating structure present for chemically produced active colloids, this does not increase the rate of reaction per active surface area at a given thickness, Figure 5d. It consequently appears that the rougher surface may be producing an increase in translational propulsion velocity that is not linked to an increase in fuel decomposition rate. Models for phoretic propulsion include velocity determining surface mobility parameters which could reasonably be expected to be modified by roughness.^38^ A recent report also observed a roughness induced speed increase for Janus micromotors, however in this case the speed increase correlated with increased catalyst turnover.^39^

It should be noted when comparing velocities that there is also a difference in surface chemistry of the inactive portion between PVD bare silica colloids and the amine‐functionalized solution prepared colloids. It has been reported that differences in chemistry which may affect hydrophobicity are only significant at larger colloid sizes (>2 µm) and if they produce significant differences in hydrophobicity (>90°).^40^ In our experiments, neither of these conditions is met. The origin of the velocity plateau for the solution‐based colloids, despite continued reactivity increases, is less obvious. This appears to indicate that a regime of behavior has been accessed where decomposition of hydrogen peroxide is no longer limiting the propulsion speed.

## 3 Conclusion

We have demonstrated a Pickering emulsion route to produce batches of active colloids which exhibit similar motility to those made by PVD. However, unlike the PVD method, the solution phase method is scalable, and does not require access to expensive equipment. Here, we made batches of active Janus particles on a small test scale producing 5 mL at ≈0.5 mg mL^−1^ mass fraction (±0.1 mg mL^−1^), however the Pickering emulsion approach can allow for gram‐scale production.^41^ The described method generates self‐motility by functionalizing silica colloids, which is advantageous due to the simplicity of producing large quantities of monodisperse colloids with selectable diameters.^23^, ^24^ Silica offers other potential advantages in controllable porosity,^25^ allowing use for drug storage and delivery which is a potential application area for active Janus particles.^26^ The methodology also leaves exposed amine groups on the catalytically inactive side of the Janus colloids, which will enable attachment of proteins, a prerequisite for the proposed mass transport applications and antibodies which could potentially allow biological recognition and specific targeting.^33^, ^34^ Despite the difference in preparation method, which is expected to produce platinum coatings with different characteristics (PVD coatings will have a gradient of thickness and reactivity from pole to equator, whereas the methods here are expected to produce a uniform coating), the observed trajectories do not reveal a significant difference in motility. This observation could help inform mechanistic understanding for active colloids. Significantly, less metal was required to produce rapid propulsion for the colloids made by Pickering masking, which is suggested to be due to the rougher, particulate platinum topography. Due to the cost of platinum, this is an additional significant benefit of the described method.

In the future, it may be possible to exert additional control over active colloids prepared using Pickering emulsion masking. For example, a large library of silanes is commercially available allowing future studies to assess the effect of different surface functional groups on the platinum morphology.^27^ We also note that there are published reports controlling the penetrative depth of the silica, known as the “Janus balance” from the one‐third submersion we observed here to approximately two‐third submersion.^42^, ^43^ This opens potential future work to explore the effect of the cap coverage on the enhanced motion at coverages <50%. We note that any such study will require the careful consideration of other factors that can alter motility such as surface roughness and the chemical composition of all surfaces of the Janus colloids. In conclusion, we hope that the Pickering emulsion route to active colloids described here will allow researchers to routinely prepare useful quantities of self‐motile colloidal, enabling more rapid development of applications for active Janus particles and investigations of predicted collective phenomena. In addition, it is hoped that solution deposition of material will open a pathway to cheaper and more biocompatible catalytic materials being used to propel motile devices, such as metal‐oxides, which have so far proven difficult to deposit by traditional PVD techniques.

## 4 Experimental Section

*Materials*: Tetraethyl orthosilane (TEOS, 98%), ammonia (25% wt), paraffin wax, sodium borohydride (99%), sodium dodecylsulfate (SDS, 98%), cetyltrimethylammonium bromide (CTAB, 98%), hexachloroplatinic acid hexahydrate (>37.5% Pt basis), hydrogen peroxide (30% wt), and methanol (99.6%) were purchased from Sigma Aldrich. APS (97%) and formaldehyde (37% wt) were purchased from Alfa Aesar. All materials were used as received. Deionized (DI) water was obtained from an Elga Purelab Option filtration system (15 MΩ cm).

*Silica Colloids*: Monodisperse silica colloids were prepared by a Stöber‐like method, based on the procedures by Nozawa et al. and Wang et al.^24^, ^44^ Briefly, a TEOS solution (1:1 in 2‐propanol, v/v %) was drip fed via syringe pump (Aladdin NE‐1000) into a magnetically stirred solution containing 2‐propanol, water, and ammonia, at a controlled rate of 2.5 mL h^−1^ at 4 °C to a final concentration of 1.24 m TEOS, 7.16 m H_2_O, and 0.141 m NH_3_OH, respectively.

The formed colloids were washed in ethanol by centrifugation and removal of the supernatant to remove excess TEOS, washed in water by centrifugation to remove ammonia (confirmed by pH) and finally washed and re‐suspended in a small amount of ethanol to remove the water and finally dried at 80 °C.

**Figure 7:**
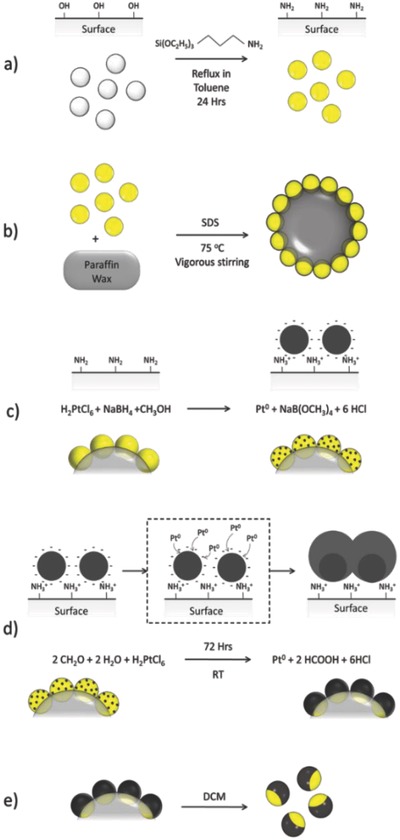
Schematic showing steps to create active Janus colloids through solution synthesis. a) Silica colloids are refluxed in toluene with APS to aminate the surface. b) The aminated silica colloids are hydrophobized with SDS and emulsified with the melted wax to form the Pickering emulsion. c) The trapped aminated silica is seeded with platinum nanoparticles. d) The seeded particles are grown using platinic acid and formaldehyde. e) The colloids with grown platinum are released using DCM to dissolve the wax.

*Silanization*: Surface functionality was controlled through self‐assembled monolayer silanization techniques which are not expected to significantly alter the size or size dispersity of the colloids. Prepared silica colloids were dispersed in toluene (2.3 wt%) by ultrasonication with excess APS (3.4 mmol m^−2^ of silica) and refluxed for 24 h. APS‐treated colloids were then washed in ethanol by centrifugation and finally dried at 80 °C, **Figure** **7**a.

*Nanoparticle Tracking Analysis*: NTA was used to obtain particle size distributions for the synthesized colloid by sampling the diffusion coefficients of a dilute dispersion of the colloids in DI water. Measurements were taken using a Malvern LM‐10 Nanosight system.

*Pickering Emulsion*: Pickering emulsions were prepared as described by Perro et al.,^22^ where bare silica colloids in conjunction with CTAB cationic surfactant are used to stabilize an emulsion of high melting point wax in its liquid state during heating through adsorption at the oil/water interface. Upon cooling the wax solidifies and traps the silica between the oil and water interface.

Due to the use of APS‐modified silica, SDS was used as an anionic surfactant to complement the colloid surface charge and hydrophobize the silica sufficiently to facilitate emulsion stabilisation.^22^

The SDS solution (1.23 × 10^−3^ m), wax, and Stöber‐like synthesized silica (10.0:1.11:0.0556 ratio by weight) were sonicated together for 15 min before heating to 75 °C and rapid stirring for 2 h, after which the stirring was stopped and the emulsion was taken off the heat and allowed to cool to room temperature solidifying the wax after which the solid phase was filtered and washed in cold methanol, Figure 7b.

*Seeding*: Platinum seeds were prepared by the rapid reduction of hexachloroplatinic acid (31.25 × 10^−6^ m) in methanol by the addition of sodium borohydride (0.066 m).

A prepared seed solution of 40 mL was then rapidly added to 0.1 g of washed Pickering emulsion powder and the solution was gently agitated over 30 min. The powder would change from a pristine white to pale gray indicating the attachment of the seeds. The seeded solid was then filtered and washed with cold methanol, Figure 7c.

*Metal Growth*: The Pt seeded embedded wax‐silica powder (0.1 g) was placed in a 1% formaldehyde solution containing water and ethanol (1:1) and hexachloroplatinic acid (at 0, 2.11, 4.23, 8.46, and 16.92 × 10^−9^ mol of platinum per cm^2^ of silica based on the starting mass of silica colloids used) and gently agitated over 72 h, Figure 7d.

*Particle Release*: The platinated particles were dispersed in dichloromethane (DCM) and centrifuged at 3000 rpm (1811 RCF) using an Eppendorf Centrifuge model 5810, to liberate the colloids from the wax mask. This was repeated three times to ensure complete removal of the wax, Figure 7. Colloids were then washed in ethanol by centrifugation to remove DCM, this was repeated three times, and then further washed in water by centrifugation to remove salts, again repeated three times. The final product was stored as an aqueous suspension.

*Colloidal Yield*: Colloidal yields, allowing quantifying any loss of colloids during the functionalization process, were obtained by microscopy observation of the number of colloids in a known volume, before and after platinum coating. To estimate colloidal volume fraction, the solution of colloids was diluted to 0.25% of the original concentration and the diluted solution was placed into a cuvette. The colloids in the cuvette were then allowed to sediment from the bulk to the bottom surface for 24 h, after which images of several areas of the bottom interface were taken and the number of colloids recorded. Conversion to volume fraction was achieved using the known 2D image area and the depth of the cuvette.

*Physical Vapor Deposition*: Platinum thin films were deposited by e‐beam evaporation of platinum metal (99.995%) using a Moorfield Minilab 80 evaporator under high vacuum (1 × 10^−9^ bar), on to glass slides containing low density arrangements of the silica colloids, prepared by spin‐coating (2000 RPM) low concentration of colloids dispersed in ethanol. Platinum thickness was measured using a quartz crystal monitor.

*2D Motion Tracking*: Platinum‐coated colloids were transferred to a hydrogen peroxide solution (15% wt), sonicated for 5 min and left standing for a further 25 min to remove trace surface contaminants, after which the solution was further diluted to 10% H_2_O_2_ by weight in which the measurements will take place.

800 × 600 resolution videos were taken for each active colloid at 33 frames s^−1^ for 30 s using a Pixelink PL‐B742F camera mounted on a Nikon eclipse inverted microscope. Multiple tracks were recorded for each set of samples (*n* = 25). A custom Labview program was used to analyze the trajectories and generate the mean squared displacement and quantify velocity.

*Reaction Rate Analysis*: Reaction rates were obtained from known concentration of active Janus colloids in 10% H_2_O_2_ under magnetic stirring.

UV–vis absorption measurements were taken at 240 nm wavelength every 30 min for a total of 180 min. The reaction rate could then be determined from the gradient using the molar extinction coefficient as stated by the manufacturer Sigma Aldrich (43.6 m ^−1^ cm^−1^).

*Platinum Thickness Analysis*: Platinum thickness was determined by calculating the sedimentation rate of the platinum‐modified colloids. The sedimentation rate was found by observing individual colloids in a dilute aqueous suspension, using a Prior scientific stage controller to log the colloidal decent over a period of 90 s. Using Stokes law, any increase to the settling velocity from the initial unmodified colloids (0.44 µm s^−1^) was attributed to the increase in average density due to the deposited platinum. The calculated additional mass of the platinum producing the density increase was converted to thickness by assuming the formation of a platinum shell covering two‐thirds of the colloid surface (as indicated by SEM).

## Conflict of Interest

The authors declare no conflict of interest.
